# Self-Assembling Conjugated Organic Materials with a Silazane Anchor Group: Synthesis, Self-Organization, and Semiconductor Properties

**DOI:** 10.3390/nano16020124

**Published:** 2026-01-16

**Authors:** Elizaveta A. Bobrova, Maxim S. Skorotetсky, Bogdan S. Kuleshov, Victoria P. Gaidarzhi, Askold A. Trul, Elena V. Agina, Oleg V. Borshchev, Sergey A. Ponomarenko

**Affiliations:** 1 Enikolopov Institute of Synthetic Polymer Materials of the Russian Academy of Sciences, Profsoyuznaya Str. 70, 117393 Moscow, Russia; 2 Moscow Center for Advanced Studies, Kulakova Str. 20, 123592 Moscow, Russia

**Keywords:** organic semiconductor, silazane group, hydrosilylation, self-organization, monolayer, Langmuir–Blodgett, Langmuir–Schaefer

## Abstract

An efficient synthetic method for the preparation of self-assembling conjugated organic materials with a silazane anchor group based on direct hydrosilylation reaction is reported. A novel organic semiconductor molecule, **NH(Si-Und-BTBT-Hex)_2_**, consisting of a polar silazane anchor group linked through undecylenic (Und) aliphatic spacers to conjugated blocks based on benzothieno[3,2-b][1]benzothiophene (BTBT) and solubilizing hexyl (Hex) end groups, was synthesized. Its self-organization on the air-water interface and solid substrates into ultrathin layers obtained by the Langmuir–Schaefer or Langmuir–Blodgett methods was investigated. Monolayer organic field-effect transistors manufactured from **NH(Si-Und-BTBT-Hex)_2_** showed operation in the *p*-type mode.

## 1. Introduction

Organic electronics is a rapidly growing field of science and technology. The development of optoelectronic devices with low cost, light weight, flexibility, and transparency has become possible due to the application of organic conjugated materials [1,2,3,4,5]. Self-assembling molecules with anchoring groups [6,7,8,9] play a special role among them, enabling the development of stable and efficient organic electronics through solution-based methods.

Organosilicon materials are a unique class of materials that combine the properties of silicon-based and organic compounds. They have potential applications in optoelectronics, as they effectively control optical properties. Owing to this ability, organosilicon materials can be employed in light-emitting devices, solar cells, and sensors. Research suggests that they can significantly improve the performance of such devices [10,11,12].

Various derivatives of benzothieno[3,2-b][1]-benzothiophene (BTBT) have attracted considerable interest for organic electronics due to their high charge carrier mobility [13,14]. Functionalization of the BTBT molecule with moieties capable of interacting with the dielectrics represents a promising strategy for enhancing interfacial properties and boosting organic field-effect transistor (OFET) performance. However, selecting a suitable functional group presents a non-trivial challenge, requiring consideration of both reactivity and material stability. The influence of chlorosilane and disiloxane groups on the organization and properties of BTBT-containing 2D nanomaterials has been investigated previously [15]. Chlorosilanes, which possess high reactivity, can covalently bond with hydroxyl-containing dielectrics [16,17], but their sensitivity to moisture complicates handling. Disiloxanes, conversely, exhibit high stability, yet their interaction with the dielectric is limited to hydrogen bonding or even van der Waals forces. Although disiloxane derivatives were previously considered less promising, recent studies have shown that optimization of film formation conditions can lead to high OFET performance [18]. Silazanes represent an intermediate option, combining moderate reactivity with stability. In this work, we aimed to improve the bonding of the organic material to the substrate by introducing a silazane anchor group capable of forming covalent bonds between silicon atoms of the self-assembling molecule and silanol groups of silicon dioxide dielectrics.

## 2. Materials and Methods

### 2.1. Materials

For the synthesis, chloro(dimethyl)silane (DMCS), platinum(0) 1,3-divynyl-1,1,3,3-tetramethyldisiloxane complex in xylene (Karstedt’s catalyst) was obtained from Sigma-Aldrich, Burlington, MA, USA. DMCS was distilled before use. Anhydrous toluene (JSC Vekton, Moscow, Russia) and THF (ECOS-1, Moscow, Russia) were distilled over CaH_2_ (JSC “Lenreaktiv”, Saint-Petersburg, Russia) under a nitrogen atmosphere before use. 1,1,3,3-tetramethyldisilazane (TMDSN) was obtained by the method described in the patent [19]. 5-(undec-10-en-1-yl)-2,2′-bithiophene [15] and 2-hexyl-(7-undec-10-en-1-yl)[1]benzothieno[3,2-b][1]benzothiophene [20] were obtained using the procedures described elsewhere.

### 2.2. Synthetic Details

*([2,2′-bithiophen]-5-yl-undecyl)chlorodimethylsilane* **(2)**: Karstedt’s catalyst (10 μL) was added to a solution of 500 mg (1.5 mmol) of 5-(undec-10-en-1-yl)-2,2’-bithiophene **(1)** and 2.84 g (30 mmol) of dimethylchlorosilane (DMCS) in anhydrous toluene (10 mL) under an atmosphere of argon, and it was stirred at 80 °C for 6 h. Toluene was removed under reduced pressure. The reaction mixture was evaporated under reduced pressure to give pure compound **2** as a colorless oily liquid (577 mg, 89%). ^1^H NMR (250 MHz, CDCl_3_) *δ*: 0.40 (s, 6H), 0.80 (t, 2H, J = 7.02 Hz), 1.24–1.41 (m, 16H), 1.68 (t, 2H, J = 7.02 Hz), 2.78 (t, 2H, J = 7.93 Hz), 6.67 (d, 1H, J = 3.67 Hz), 6.98 (t, 2H, J = 3.66 Hz), 7.09 (d, 1H, J = 2.44 Hz), 7.17 (d, 1H, J = 5.19 Hz).

*1,3-bis([2,2′-bithiophen]-5-yl-undecyl)-1,1,3,3-tetramethyldisiloxane*: ^1^H NMR (250 MHz, CDCl_3_) *δ*: 0.40 (s, 12H), 0.81 (m, 4H), 1.21–1.37 (m, 32H), 1.68 (t, 2H, J = 7.32 Hz), 2.78 (t, 4H, J = 7.63 Hz), 6.67 (d, 2H, J = 3.48 Hz), 6.98 (d, 2H, J = 3.66 Hz), 7.00 (d, 2H, J = 3.49 Hz), 7.09 (d, 2H, J = 3.49 Hz), 7.16 (d, 2H, J = 5.14 Hz), ^29^Si NMR (60 MHz, CDCl_3_) *δ*: 7.34.

*Bis(([2,2′-bithiophen]-5ylundecyl)(dimethylsilyl)amine* **HN(Si-Und-2T)_2_**: Karstedt’s catalyst (10 μL) was added to a solution of 500 mg (1.5 mmol) of 5-(undec-10-en-1-yl)-2,2′-bithiophene (**1**) and 93 mg (0.7 mmol) of 1,1,3,3-tetramethylsilazane (TMDSN) in anhydrous toluene (10 mL) under an atmosphere of argon, and it was stirred at 80 °C for 6 h. Toluene was removed under reduced pressure. The reaction mixture was evaporated under reduced pressure and purified by preparative chromatography in THF as an eluent to give pure compound **HN(Si-Und-2T)_2_** (450 mg, 90%), ^1^H NMR (250 MHz, CDCl_3_) *δ*: 0.25 (d, 6H, J = 3.19), 0.67–0.80 (m, 4H), 1.20–1.37 (m, 32H), 1.53 (s, 1H), 1.61–1.73 (m, 4H), 2.78 (t, 4H, J = 7.48 Hz), 6.66 (d, 2H, J = 3.62 Hz), 6.95–7.00 (m, 4H), 7.08 (d, 2H, J = 3.62 Hz), 7.15 (d, 2H, J = 5.07 Hz), 7.30–7.36 (m, 6H), 7.51–7.57 (m, 4H).

*Bis((11-(7-hexyl)[1]benzothieno[3,2-b][1]-benzothiophen-2-yl)undecyl)dimethylsilyl)amine* **HN(Si-Und-BTBT-Hex)_2_**: Karstedt’s catalyst (10 μL) was added to a solution of 500 mg (1.0 mmol) of 2-hexyl-7-(undec-10-en-1-yl)[1]benzothieno[3,2-b][1]benzothiophene and 67 mg (0.5 mmol) of TMDSN in anhydrous toluene (10 mL) under an atmosphere of argon, and it was stirred at 80 °C for 6 h. Toluene was removed under reduced pressure. The reaction mixture was evaporated under reduced pressure and purified by preparative chromatography in THF as an eluent to give pure compound **HN(Si-Und-BTBT-Hex)_2_** (380 mg, 70%), ^1^H NMR (250 MHz, CDCl_3_) *δ*: 0.00–0.07 (m, 12H), 0.43–0.54 (m, 4H), 0.86–0.93 (m, 6H), 1.24–1.41 (m, 44H), 1.53 (s, 1H), 1.62–1.75 (m, 8H), 2.70–2.78 (m, 8H), 7.25 (d, 4H, J = 8.26 Hz), 7.71 (t, 8H, J = 8.12 Hz); ^13^C NMR (75 MHz, CDCl_3_*δ*: −0.78, 14.07, 19.03, 22.59, 23.72, 28.98, 29.33, 29.41, 29.54, 29.61, 29.71, 31.65, 31.69, 31.73, 33.60, 36.12, 121.03, 123.29, 125.80, 131.20, 132.54, 140.04, 142.42; ^29^Si NMR (60 MHz, CDCl_3_) *δ*: 3.35. MS-MALDI (*m*/*z*): [M]+ calcd for (C_66_H_95_NS_4_Si_2_) 1057.6111, found 1057.6108.

### 2.3. Organic Field-Effect Transistor Preparation

Organic field-effect transistors (OFETs) with semiconducting layers based on LB and LS films were fabricated on highly doped silicon substrates with 300 nm thermally grown oxide. Gold source and drain electrodes were thermally evaporated through a shadow mask. The bottom-contacts/bottom-gate OFET configuration had a fixed channel width (W) of 1000 μm and length (L) of 30 μm. No additional dielectric treatment was applied before deposition of the organic semiconducting layer. Electrical measurements were carried out on a probe station (ProbeStation 100, Printeltech LLC, Moscow, Russia) using a Keithley 2634B source-measure unit (Tektronix, Beaverton, OR, USA) in air at room temperature. Saturated field-effect mobilities were extracted from transfer characteristics using the Shockley gradual channel model [21]:

(1) $$I_{d}=\frac{W}{2L}\mu_{sat}C_{i}{\left(V_{g}-V_{th}\right)}^{2}$$

where *μ*_*sat*_ is the charge carrier mobility in the saturated regime, *V*_*g*_ is the gate voltage, *V*_*t*ℎ_ is the threshold voltage, and *C*_*i*_ = 11.5 nF cm^−2^ is the dielectric capacitance calculated from the oxide thickness and dielectric constant.

## 3. Results and Discussion

### 3.1. Synthesis

**Scheme 1:**
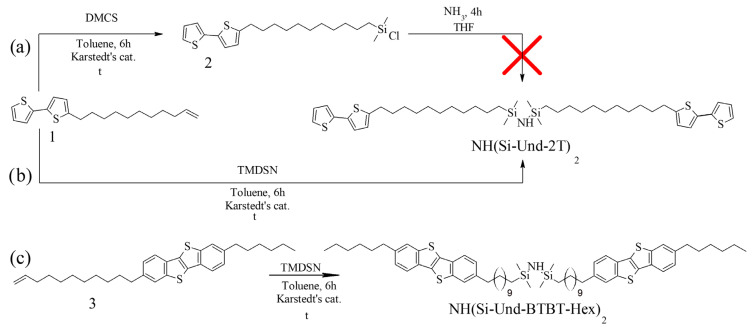
Synthesis of **HN(Si-Und-2T)_2_** (**a**,**b**) and **HN(Si-Und-BTBT-Hex)_2_** (**c**).

Laboratory and industrial synthesis of silazanes is typically based on the ammonolysis of chlorosilanes [22,23,24,25], which involves passing ammonia gas through a solution of chlorosilanes. To test the synthesis, a model compound with an aliphatic spacer and a bithiophene conjugated core—(*bis*(([2,2′-bithiophen]-5-yl-undecyl)(dimethylsilyl) amine) **HN(Si-Und-2T)_2_**—was proposed (Scheme 1a,b). To obtain the desired structure, it was necessary to synthesize the corresponding chlorosilane precursor—[2,2′-bithiophen]-5-yl-undecyl)chlorodimethylsilane **(2)**. For this purpose, a hydrosilylation reaction of 5-(undec-10-en-1-yl)-2,2′-bithiophene (**1**) with dimethylchlorosilane (DMCS) using Karstedt’s catalyst was carried out with 89% yield of chlorosilane **2** (Scheme 1a). However, the ammonolysis reaction did not produce the desired structure. Instead, it resulted in the formation of a siloxane dimer, as was confirmed by H^1^ and Si^29^ NMR spectra (see Electronic Supplementary Materials Figures S2 and S3) and their comparison with those of *bis*(([2,2′-bithiophen]-5-yl-undecyl)dimethylsiloxane [26].

Because direct ammonolysis of chlorosilane **2** with ammonia was unsuccessful, another route was proposed. Compound **1** and 1,1,3,3-tetramethyldisilazane (**TMDSN**) were reacted by hydrosilylation in the presence of Karstedt’s catalyst [27] to form the desired silazane dimer, **HN(Si-Und-2T)_2_** (Scheme 1b). The reaction yield, according to GPC analysis, was 92%.

After testing and optimizing the synthesis conditions on the model compounds with a bithiophene conjugated core, we proceeded to synthesize the target molecule with a conjugated BTBT core—*bis*((11-(7-hexyl)[1]benzothieno[3,2-b][1]-benzothiophen-2-yl)undecyl)dimethylsilyl)amine **HN(Si-Und-BTBT-Hex)_2_**. To obtain it, the hydrosilylation reaction of the corresponding precursor—2-hexyl-7-(undec-10-en-1-yl)[1]benzothieno[3,2-b][1]benzothiophene (**3**)—can be carried out either in two steps (first obtaining a monosubstituted product and then reacting it with a second portion of the unsaturated starting material, as shown in work [15]) or in a single step with a double excess of alkene.

The one-stage process is complicated by the fact that, upon heating, some of the **TMDSN** may leave the reaction mixture and condense on the vessel walls. On the other hand, the two-step process requires the use of an excess of silazane and its careful removal through evacuation. Additionally, the monosubstituted silazane appears less hydrolytically stable, and the second hydrolysis step occurs with a low yield. As a result, in practice, the single-step approach has been proven more effective, allowing the production of the target silazane with a yield of 70% (see Scheme 1c).

Thus, the desired silazanes can be obtained by reacting **TMDSN** with a corresponding alkene precursor of the required structure through a hydrosilylation reaction. This approach allows for a high-yield product using small amounts of the starting materials.

### 3.2. The Thermal Stability and Phase Behavior

**Figure 1:**
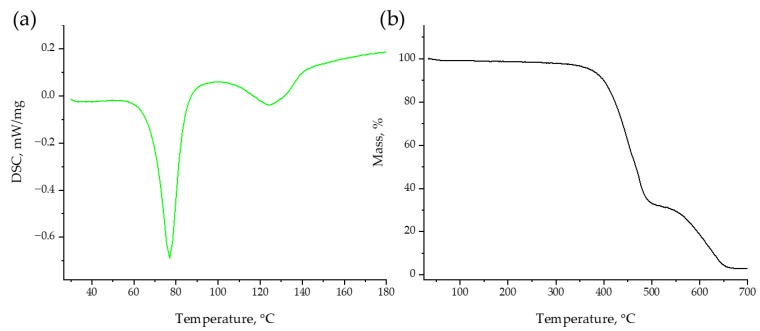
First heating DSC curve for **NH(Si-Und-BTBT-Hex)_2_** (**a**); TGA of **HN(Si-Und-BTBT-Hex)_2_** in the air (**b**).

**Figure 2:**
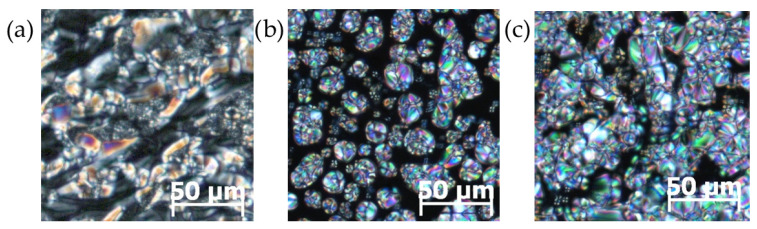
Optical microphotographs of a thick film of **N(Si-Und-BTBT-Hex)_2_** in crossed polarizers at room temperature (23 °C) (**a**), at 125 °C (cooling) (**b**), and at 90 °C (cooling) (**c**).

The thermal stability and phase behavior of **HN(Si-Und-BTBT-Hex)_2_** were investigated by differential scanning calorimetry (DSC—Figure 1a), thermogravimetric analysis (TGA—Figure 1b), and polarizing optical microscopy (POM—Figure 2). The original data for TGA and DSC (1st heating, cooling, and 2nd heating) are given in Figure S9 in the Electronic Supplementary Materials. According to TGA data, the silazane dimer is stable in air at temperatures up to 370 °C. DSC data show that silazane dimer **HN(Si-Und-BTBT-Hex)_2_** shows two enantiotropic first-order phase transitions, with peaks at 77 °C and 125 °C and enthalpies of 30.45 J g^−1^ and 12.9 J g^−1^, respectively. Optical polarizing microscopy showed the formation of fan-shaped textures characteristic of smectic A mesophase SmA (Figure 2). These results match well with the phase behavior of a similar siloxane dimer, **O(Si-Und-BTBT-Hex)_2_**, for which melting and isotropization temperatures are 83 °C and 144 °C, with the corresponding enthalpies of 35 J g^−1^ and 18 J g^−1^, respectively [15]. However, later detailed SAXS/WAXS measurements allowed us to attribute the low-temperature phase of **O(Si-Und-BTBT-Hex)_2_** to ordered smectic E mesophase SmE [20]. Bearing in mind the similarity of the chemical structures and enthalpies of phase transitions for **HN(Si-Und-BTBT-Hex)_2_** and **O(Si-Und-BTBT-Hex)_2_** dimers, it can be suggested that the low-temperature phase for the silazane dimer should also be SmE rather than a crystal phase, which, however, requires further experimental confirmations.

### 3.3. Films

**Figure 3:**
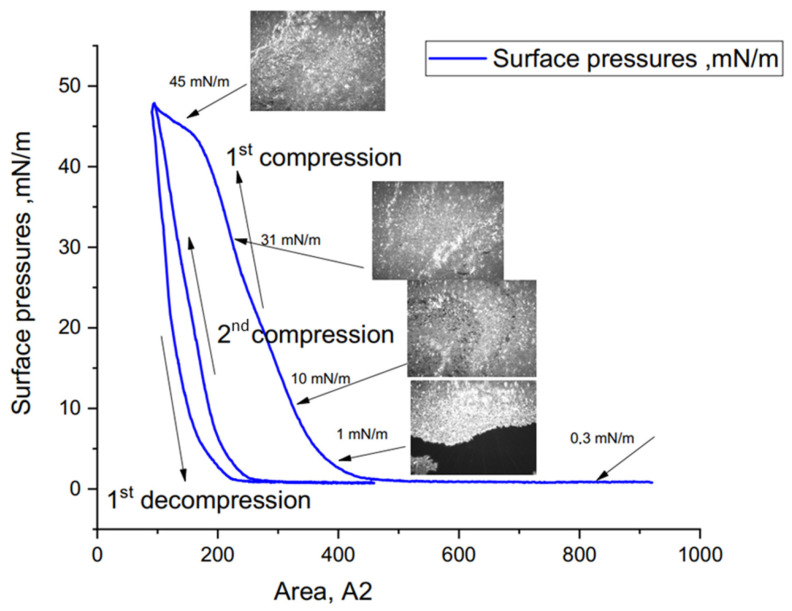
Langmuir isotherms for silazane dimer **HN(Si-Und-BTBT-Hex)**_2_ (compression-decompression-compression cycle) with the Brewster angle microscopy images obtained at the indicated surface pressures.

**Figure 4:**
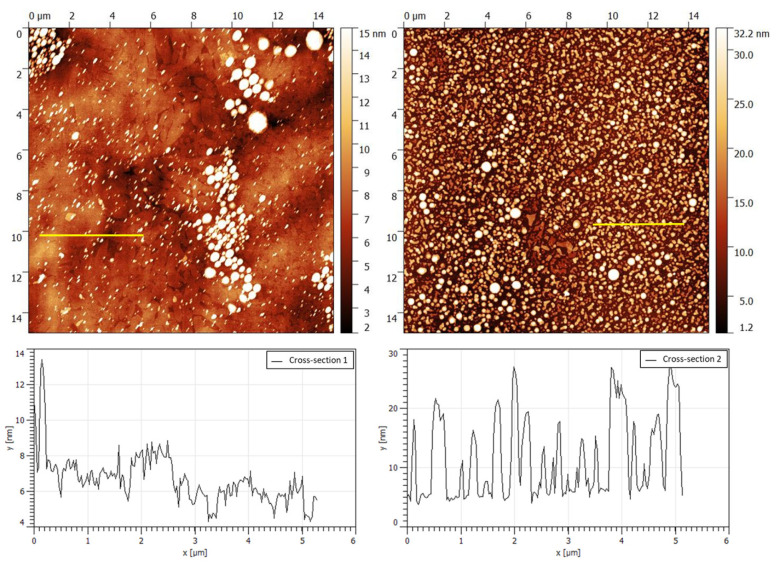
AFM images of the LS (**left**) and LB (**right**) films of silazane dimer **HN(Si-Und-BTBT-Hex)_2_**. Yellow lines show cross-sections, the images of which are shown below each AFM image.

Langmuir films of the synthesized compound **HN(Si-Und-BTBT-Hex)_2_** were obtained on a water surface and then were transferred to silicon substrates using Langmuir–Blodgett (LB) or Langmuir–Schaefer (LS) techniques. The film transfer was performed under the first compression at a surface pressure of 40 mN m^−1^, corresponding to a fully compressed layer before collapse (Figure 3). The balance between the hydrophilic silazane group and the hydrophobic BTBT fragment in **HN(Si-Und-BTBT-Hex)_2_** enables the formation of Langmuir layers on both water surfaces and silicon substrates. The measured thickness of the bottom-lying sublayer was approx. 2 nm, about 40% less than the calculated molecule length in its closed conformation. The full calculated molecule length in its extended conformation is 6.6 nm. Such a thickness should correspond to a monolayer with tilted BTBT fragments. Atomic force microscopy (AFM) data show that LB films have a more heterogeneous morphology than LS films, due to transfer technique differences and the tendency of the monolayer to collapse during the LB transfer (Figure 4).

### 3.4. OFETs

**Figure 5:**
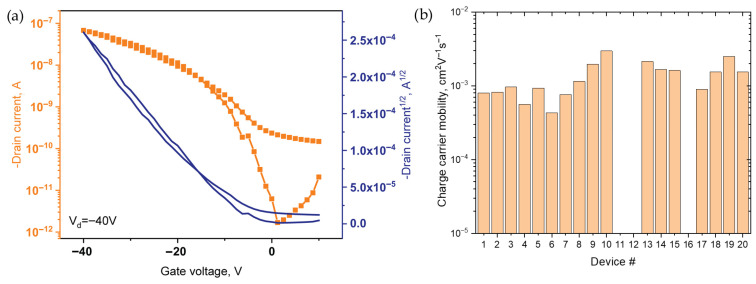
A typical transfer curve (**a**) and charge carrier mobility distribution (**b**) for the LS OFET based on silazane dimer **HN(Si-Und-BTBT-Hex)_2_**.

OFETs demonstrated typical *p*-type behavior with different operating device yields depending on the fabrication technique. The yield for LS was from 85 to 95% (from 17 to 19 of 20 devices were working). A typical transfer curve of the LS OFET based on silazane dimer **HN(Si-Und-BTBT-Hex)_2_** is shown in Figure 5a, and overall characteristics are summarized in Table 1. The maximum field-effect mobility was 3.0 × 10^−3^ cm^2^V^−1^s^−1^, with an average value of 1.4 × 10^−3^ cm^2^V^−1^s^−1^ (charge carrier mobility variation from device to device is shown in Figure 5b). The average values of the threshold voltage and on/off ratio were found to be −8 V and 10^4^–10^5^, respectively. It is worth mentioning that batch-to-batch reproducibility was pretty good since the variation in OFETs key parameters was pretty low from batch to batch.

The electrical performance of the LB OFET was quite poor, with a device yield of 20–40% (only 4–8 of the 20 tested devices demonstrated field effect with charge carrier mobility above 1 × 10^−5^ cm^2^V^−1^s^−1^). A typical transfer curve of the LB OFET based on silazane dimer **HN(Si-Und-BTBT-Hex)_2_** is shown in Figure S10 (see Electronic Supplementary Materials Figure S10). The average values of charge carrier mobility, threshold voltage, and on/off ratio were found to be 4.5 × 10^−4^ cm^2^/Vs, −12V, and 10^3^, respectively. Batch-to-batch reproducibility was also poor, which can be explained by film cracking during transfer from the water surface via the LB technique (see Electronic Supplementary Materials Figure S11). Thus, one can conclude that the LB technique is not suitable for device fabrication.

Comparison of the device prepared from solutions with different concentrations demonstrated that, on the one hand, for LS OFETs, charge carrier mobility has a maximum at the dimer concentration of 0.5 gL^−1^, while decreasing or increasing the concentration leads to electrical performance decreasing. On the other hand, LB OFETs somehow operate only when based on the layers obtained at a concentration of 0.33 gL^−1^. These data were compared with the data obtained for the devices prepared under similar conditions from siloxane dimer **O(Si-Und-BTBT-Hex)_2_** containing the same **Und-BTBT-Hex** chains but tetramethyldisiloxane instead of tetramethyldisilazane central unit responsible for interactions with water on the air-water interface [28]. It is easy to see that the best electrical performance of the siloxane dimer is higher (the average charge carrier mobility was 0.02 cm^2^V^−1^s^−1^); however, it is worth mentioning that the very first publication for this dimer also demonstrated lower performance (0.003 cm^2^V^−1^s^−1^) [15], and only after great improvement (including an additional PMMA layer) did the charge carrier mobility reach 0.47 cm^2^V^−1^s^−1^ with the average value of 0.16 cm^2^V^−1^s^−1^ [18]. Here, only the data for the ODMS-treated substrate is shown for a clearer comparison. ODMS was not used in the case of **HN(Si-Und-BTBT-Hex)_2_**, since it prevents good interaction between the silazane dimer and substrate. It is also interesting that comparing LB OFETs from **O(Si-Und-BTBT-Hex)_2_** and **HN(Si-Und-BTBT-Hex)_2_**, it is clear that increasing the solution concentration leads to OFET performance decreasing up to complete loss of field-effect, while for LS OFETs, the behavior is different. As was mentioned for **HN(Si-Und-BTBT-Hex)_2_**, charge carrier mobility has a maximum at the dimer concentration of 0.5 gL^−1^. At the same time, siloxane-based LS OFET performance is decreasing with concentration increasing (maximum performance achieved for a concentration of 0.33 gL^−1^). To explain such behavior, more investigations are required.

It is worth noting that monolayer OFETs can be obtained from various organic semiconductor (**OSC**) materials by different techniques [29]. Usually, very slow vacuum sublimation of non-functional OSC gives higher field-effect mobility due to their better ordering. Solution processing of non-functional molecules also leads to high field-effect mobility of mono- or ultrathin layer OFETs [30]. However, this method does not allow covering all the surface of the substrate with the functional layer—only separate monolayer single crystals can be obtained [31]. The advantages of Langmuir techniques are their ease and fast processing of monolayer films and optoelectronic devices based on them [32]. Thus, Chi Kin Lo et al. [33] employed LB technology to fabricate highly ordered thin films of amphiphilic molecules based on diketopyrrolopyrrole (DPP) cores (DPP-A and DPP-S with one or two 2-(2-(2-methoxyethoxy)ethoxy)ethyl chains, respectively), achieving edge-on molecular orientation. The hole mobility values obtained for these monolayer films in OFET devices were on the order of 10^−4^ cm^2^V^−1^s^−1^, with an I_on_/I_off_ ratio of 10^2^. Previously, a molecular perylenediimide-based LB monolayer OFET device yielded an electron carrier mobility as high as 10^−2^ cm^2^V^−1^s^−1^ and an I_on_/I_off_ ratio of 10^4^ [34]. Therefore, the results obtained are in line with the literature data on the other monolayer organic semiconductor materials prepared by Langmuir techniques. Nevertheless, novel self-assembling organic semiconductors **HN(Si-Und-BTBT-Hex)_2_** have high potential for further optimization of their performance in monolayer OFETs due to the intrinsic properties of the BTBT semiconductor core and good self-assembling properties of the silazane anchor groups.

## 4. Conclusions

In conclusion, an efficient methodology for the synthesis of novel functional organic semiconductors featuring an anchoring silazane group capable of self-organization at the water-air interface has been developed. Two approaches—hydrosilylation of alkenes and direct amination of chlorosilanes—were examined. As a result, the novel self-assembling organic semiconductor **HN(Si-Und-BTBT-Hex)_2_**, comprising a polar silazane group linked through flexible undecylenic spacers to BTBT-based conjugated blocks, was synthesized. Its self-organization in the water-air interface was investigated, and OFETs operating in *p*-type mode were fabricated, with LS films showing better electrical characteristics than LB films. LS OFETs based on **HN(Si-Und-BTBT-Hex)_2_** outperform similar devices obtained from siloxane dimer **O(Si-Und-BTBT-Hex)_2_** at higher concentrations, 0.5 and 1.0 g L^−1^, but are inferior in performance for devices prepared at lower concentrations, 0.33 g L^−1^. Therefore, the novel organic semiconductor molecule **NH(Si-Und-BTBT-Hex)_2_**, consisting of a polar silazane anchor group, is very promising, and further optimization of OFET fabrication is expected to improve its field-effect mobility.

## Figures and Tables

**Table 1:** Overall characteristics for the LS/LB OFETs based on silazane dimer **HN(Si-Und-BTBT-Hex)_2_** and siloxane dimer **O(Si-Und-BTBT-Hex)_2_**.

Compound	Batch	DM	C, g L^−1^	*µ_max_* (*µ_ave_*), cm^2^V^−1^s^−1^	*V_th_*, V	*I_on_*_/*off*_
**HN(Si-Und-BTBT-Hex)_2_**	1	LB	0.33	5.3 × 10^−4^ (4.5 × 10^−4^)	−10 to −15	10^3^–10^4^
	2	LB	0.5	-	-	-
	3	LB	1	-	-	-
	4	LS	0.33	5.0 × 10^−4^ (3.6 × 10^−4^)	−12 to −4	10^3^–10^4^
	5	LS	0.5	3.0 × 10^−3^ (1.4 × 10^−3^)	−10 to −7	10^4^–10^5^
	6	LS	1	1.5 × 10^−3^ (4.9 × 10^−4^)	−10 to −2	10^3^–10^5^
**O(Si-Und-BTBT-Hex)_2_** *	7	LB	0.33	1.0 × 10^−3^ (7.0 × 10^−4^)	−5 to 0	10–10^4^
	8	LB	0.5	9.0 × 10^−4^ (3.0 × 10^−4^)	−15 to −10	10–10^3^
	9	LB	1	-	-	-
	10	LS	0.33	7.0 × 10^−2^ (2.0 × 10^−2^)	−7 to +5	10–10^5^
	11	LS	0.5	1.0 × 10^−3^ (5.0 × 10^−4^)	−10 to −5	10–10^3^
	12	LS	1	1.0 × 10^−6^ (1.0 × 10^−6^)	−35 to −40	10

Notes: Batch—single experiment on the substrate with 20 devices each. DM—deposition method, C—concentration, *µ_max_* (*µ_ave_*)—maximum (average) charge carrier mobility, *V_th_*—threshold voltage, I_on/off_—on/off current ratio. * Data for siloxane dimer **O(Si-Und-BTBT-Hex)_2_** taken from ref. [28].

## Data Availability

The data presented in this study are available on request from the corresponding authors.

## Supplementary Materials

The following supporting information can be downloaded at https://www.mdpi.com/article/10.3390/nano16020124/s1, General methods used, Figure S1: ^1^H NMR spectrum of compound **2** in CDCl_3_; Figure S2: ^1^H NMR spectrum of 1,3-*bis*([2,2′-bithiophen]-5-ylundecyl)-1,1,3,3-tetramethyldisiloxane in CDCl_3_; Figure S3: ^29^Si NMR spectrum of 1,3-bis([2,2′-bithiophen]-5-yl-undecyl)-1,1,3,3-tetramethyldisiloxane in CDCl_3_; Figure S4: ^1^H NMR spectrum of **NH(Si-Und-2T)_2_** in CDCl_3_; Figure S5: ^1^H NMR spectrum of **N(Si-Und-BTBT-Hex)_2_** in CDCl_3_; Figure S6: ^13^C NMR spectrum of HN(Si-Und-BTBT-Hex)_2_ in CDCl_3_; Figure S7: ^29^Si NMR spectrum of **HN(Si-Und-BTBT-Hex)_2_** in CDCl_3_; Figure S8: MALDI-TOF spectra of **HN(Si-Und-BTBT-Hex)_2_**; Figure S9: TGA, DSC of **HN(Si-Und-BTBT-Hex)_2_**, Figure S10: A typical transfer curve (a) and charge carrier mobility distribution (b) for the LB OFET based on silazane dimer **HN(Si-Und-BTBT-Hex)_2_**, Figure S11: A film cracking during transfer from water surface via LB technique, Figure S12: Langmuir isotherms for silazane dimer HN(Si-Und-BTBT-Hex)_2_.

## Abbreviations

The following abbreviations are used in this manuscript:

BTBT	Benzothieno[3,2-b][1]benzothiophene
OFETs	Organic field-effect transistors
DSC	Differential scanning calorimetry
POM	Polarizing optical microscopy
TGA	Thermogravimetric analysis
TMDSN	1,1,3,3-tetramethylsilazane
GPC	Gel permeation chromatography
THF	Tetrahydrofuran
LB	Langmuir–Blodgett
LS	Langmuir–Schaefer
DMCS	Dimethylchlorosilane
AFM	Atomic force microscopy
