# Supplementary material for: Self-Assembling Conjugated Organic Materials with a Silazane Anchor Group: Synthesis, Self-Organization, and Semiconductor Properties
Source: Nanomaterials (Basel). 2026 Jan 16;16(2):124. doi: 10.3390/nano16020124 (PMC12844271; doi:10.3390/nano16020124)
Supplement: Supplementary file 1 [file nanomaterials-16-00124-s001.zip › nanomaterials-4041692-supplementary.pdf]

Electronic supplementary materials

Self-assembling conjugated organic materials with a silazane anchor group: synthesis, self-organization and semiconductor properties

Elizaveta A. Bobrova[1,2], Maxim S. Skorotetcky[1], Bogdan S. Kuleshov[1], Victoria P. Gaidarzhi[1], Askold A. Trul[1], Elena V. Agina[1], Oleg V. Borshchev[1]*, Sergey A. Ponomarenko[1]*

[1] Enikolopov Institute of Synthetic Polymer Materials of the Russian Academy of Sciences,

Profsoyuznaya Str. 70, Moscow 117393, Russia.

[2] Moscow Institute of Physics and Technology, 141700, Institutsky lane 9, Dolgoprudny, Moscow region.

[*] Correspondence: e-mail: borshchev@ispm.ru ; ponomarenko@ispm.ru, tel: +7 (495)3325897

Contents:

1. General methods used S2
2. Figure S1. ^{1}H NMR spectrum of compound **2** in $CDCl_3$ S3
3. Figure S2. ^{1}H NMR spectrum of 1,3-*bis*([2,2'-bithiophen]-5-ylundecyl)-1,1,3,3-tetramethyl-disiloxane in $CDCl_3$. S3
4. Figure S3. ^{29}Si NMR spectrum of 1,3-*bis*([2,2'-bithiophen]-5-yl-undecyl)-1,1,3,3-tetramethyl-disiloxane in $CDCl_3$. S4
5. Figure S4. ^{1}H NMR spectrum of **NH(Si-Und-2T)$_2$** in $CDCl_3$. S4
6. Figure S5. ^{1}H NMR spectrum of **N(Si-Und-BTBT-Hex)$_2$** in $CDCl_3$ S5
7. Figure S6. ^{13}C NMR spectrum of **HN(Si-Und-BTBT-Hex)$_2$** in $CDCl_3$ S5
8. Figure S7. 29Si NMR spectrum of **HN(Si-Und-BTBT-Hex)$_2$** in $CDCl_3$ S6
9. Figure S8. MALDI-TOF spectra of **HN(Si-Und-BTBT-Hex)$_2$** S6
10. Figure S9. TGA, DSC of **HN(Si-Und-BTBT-Hex)$_2$** S7
11. Figure S10. A typical transfer curve (a) and charge carrier mobility distribution (b) for the LB OFET based on silazane dimer **HN(Si-Und-BTBT-Hex)$_2$** S7
12. Figure S11. A film cracking during transfer of silazane dimer HN(Si-Und-BTBT-Hex)$_2$ from water surface via LB technique S8
13. Figure S12. Langmuir isotherms for silazane dimer HN(Si-Und-BTBT-Hex)$_2$ (compression-decompression-compression cycle) with the Brewster angle microscopy images obtained at the indicated surface pressures S9

General methods used

^1H NMR spectra were recorded in a "Bruker WP-250" SY spectrometer, working at a frequency of 250.13 MHz and utilizing $CDCl_3$ signal (7.25 ppm) as the internal standard. ^{13}C and ^{29}Si NMR spectra were recorded using a "Bruker Avance II 300" spectrometer at 75 and 60 MHz, respectively. In the case of ^1H NMR spectroscopy, the compounds to be analyzed were taken in the form of 1% solutions in $CDCl_3$. In the case of ^{13}C and ^{29}Si NMR spectroscopy, the compounds to be analyzed were taken in the form of 5% solutions in $CDCl_3$. The spectra were then processed on the computer using the ACD Labs software.

GPC analysis was performed by means of a Shimadzu LC10AVP series chromatograph (Japan) equipped with an RID-10AVP refractometer and SPD-M10AVP diode matrix as detectors and a Phenomenex column (USA) with a size of 7.8×300 mm^2 filled with the Phenogel sorbent with a pour size of 500 Å; THF was used as the eluent. For thin layer chromatography (TLC), "Sorbfil" (Russia) TLC plates were used. In the case of column chromatography, silica gel 60 (Merck, Germany) was taken.

Elemental analysis of C, H elements was carried out using CHN automatic analyzer CE1106 (Italy). Experimental error is 0.30−0.50%. The burning was done in the Sheninger flask using alkaline solution of hydrogen peroxide as an absorbent. Experimental error is 0.30−0.50%. The settling titration using $BaCl_2$ was applied to analyze sulfur. Spectrophotometry technique was used for the Si analysis.

Thermogravimetric analysis was carried out in dynamic mode in 30–900 °C interval using Mettler Toledo TG50 (USA) system equipped with M3 microbalance allowing measuring the weight of samples in 0–150 mg range with 1 µg precision. Heating rate was 10 °C min^{-1}. Every compound was studied twice: in air and in nitrogen flow of 200 mL min^{-1}. DSC scans were obtained with Mettler Toledo DSC30 (USA) system with 10 °C min^{-1} heating/cooling rate in temperature range of +20–250 °C. Nitrogen flow of 50 mL min^{-1} was used.

Self-assembly of the materials synthesized on the air-water interface was investigated by Langmuir technique. The spreading solution was prepared by dissolving the materials in toluene at the concentration of 0.33 g L^{-1}. The solution was spread on the water surface with a microsyringe, and the film was then left for 5 min to equilibrate before the compression started. Data were collected with a Nima 712BAM system (UK) using a Teflon trough and barriers at room temperature. The monolayers were compressed with the speed equal to 100 mm min^{-1}, Langmuir films were obtained by transfer on silicon substrates with gold contacts. The vertical (LB) or horizontal (LS) dipping methods with a dipping speed of 8 mm min^{-1}, were used to obtain monolayer films. Film transfers were performed at different surface pressures close but before to the collapse point.

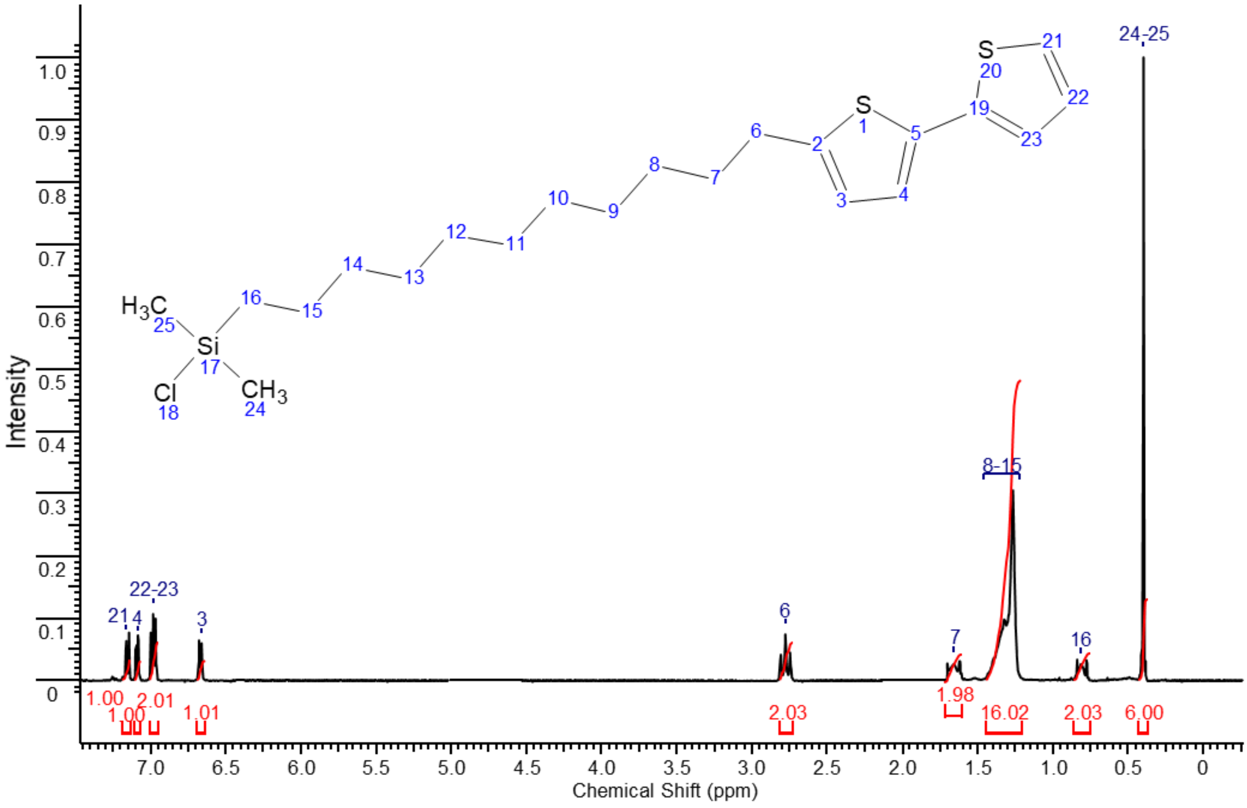

Figure S1. ^{1}H NMR spectrum of compound **2** in $CDCl_3$.

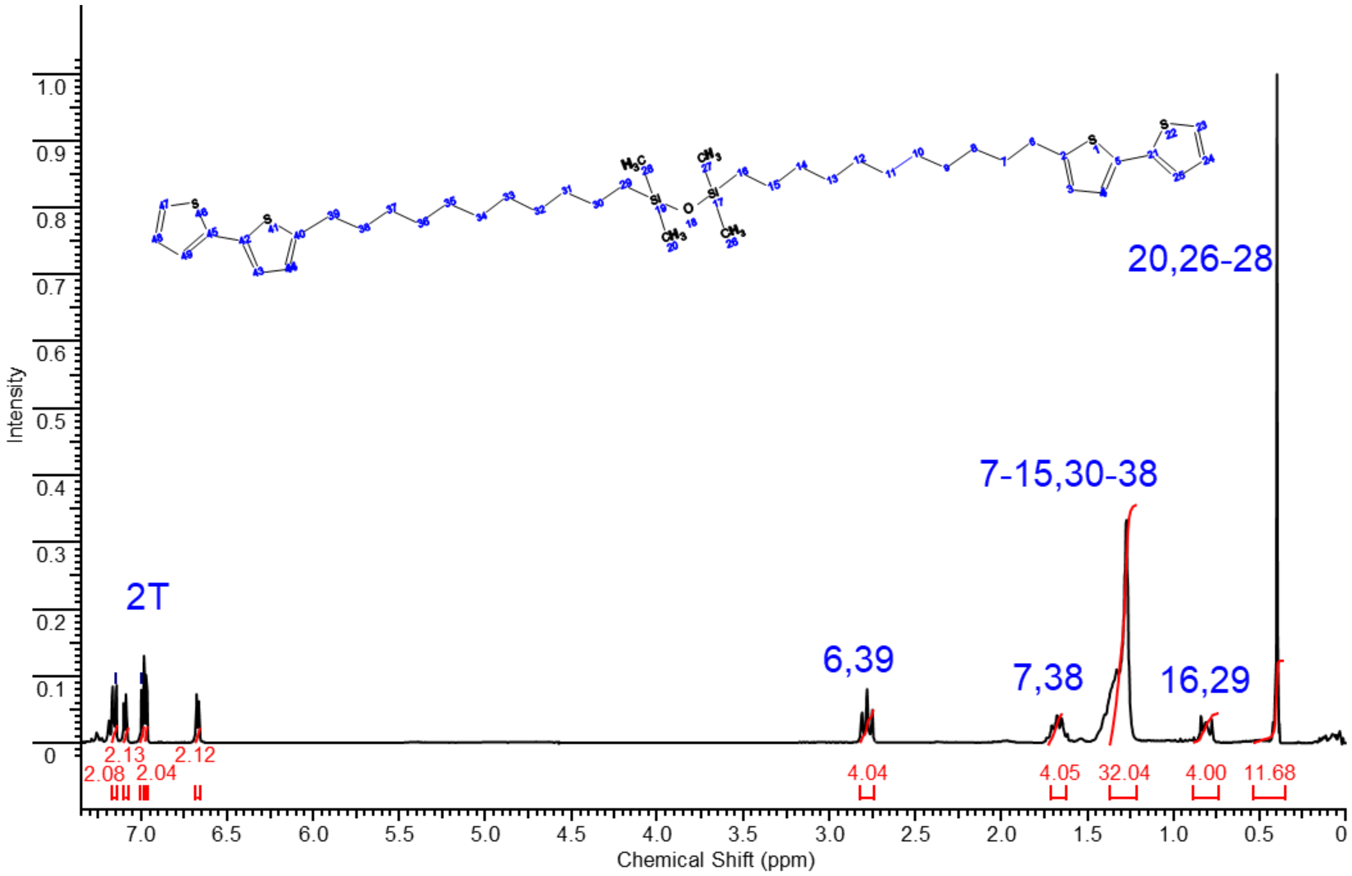

Figure S2. ^{1}H NMR spectrum of 1,3-*bis*([2,2'-bithiophen]-5-ylundecyl)-1,1,3,3-tetramethyldisiloxane in $CDCl_3$.

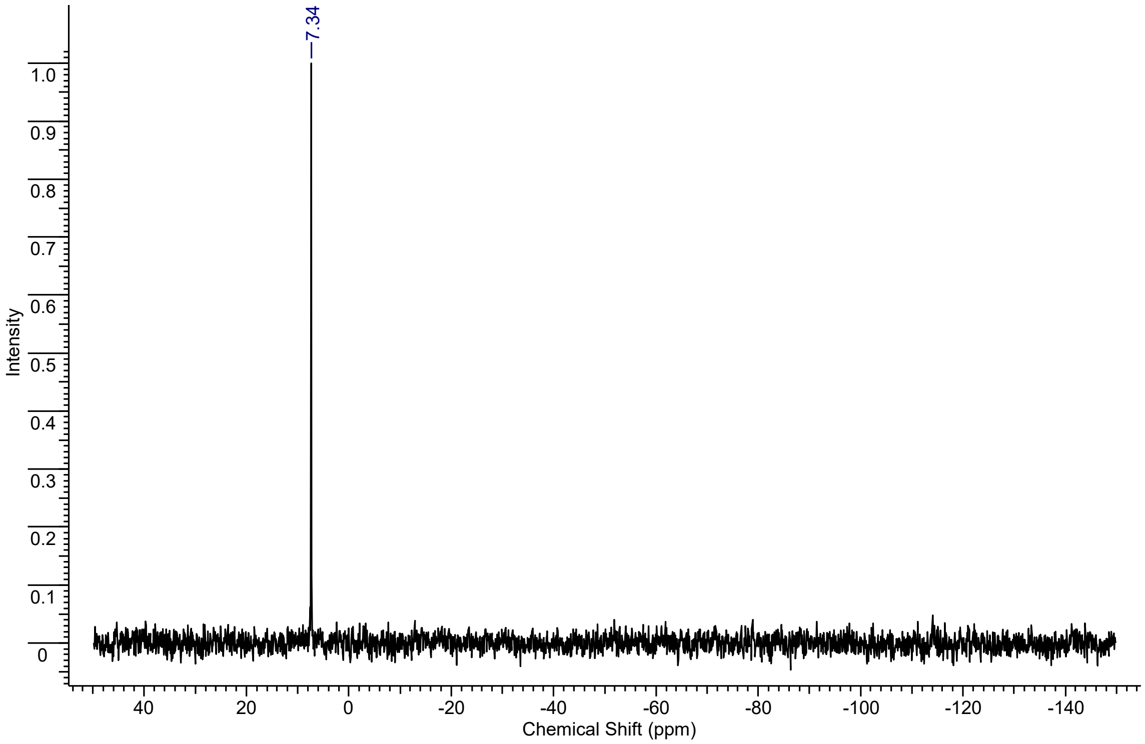

Figure S3. ^{29}Si NMR spectrum of 1,3-*bis*([2,2'-bithiophen]-5-yl-undecyl)-1,1,3,3-tetramethyldisiloxane in $CDCl_3$.

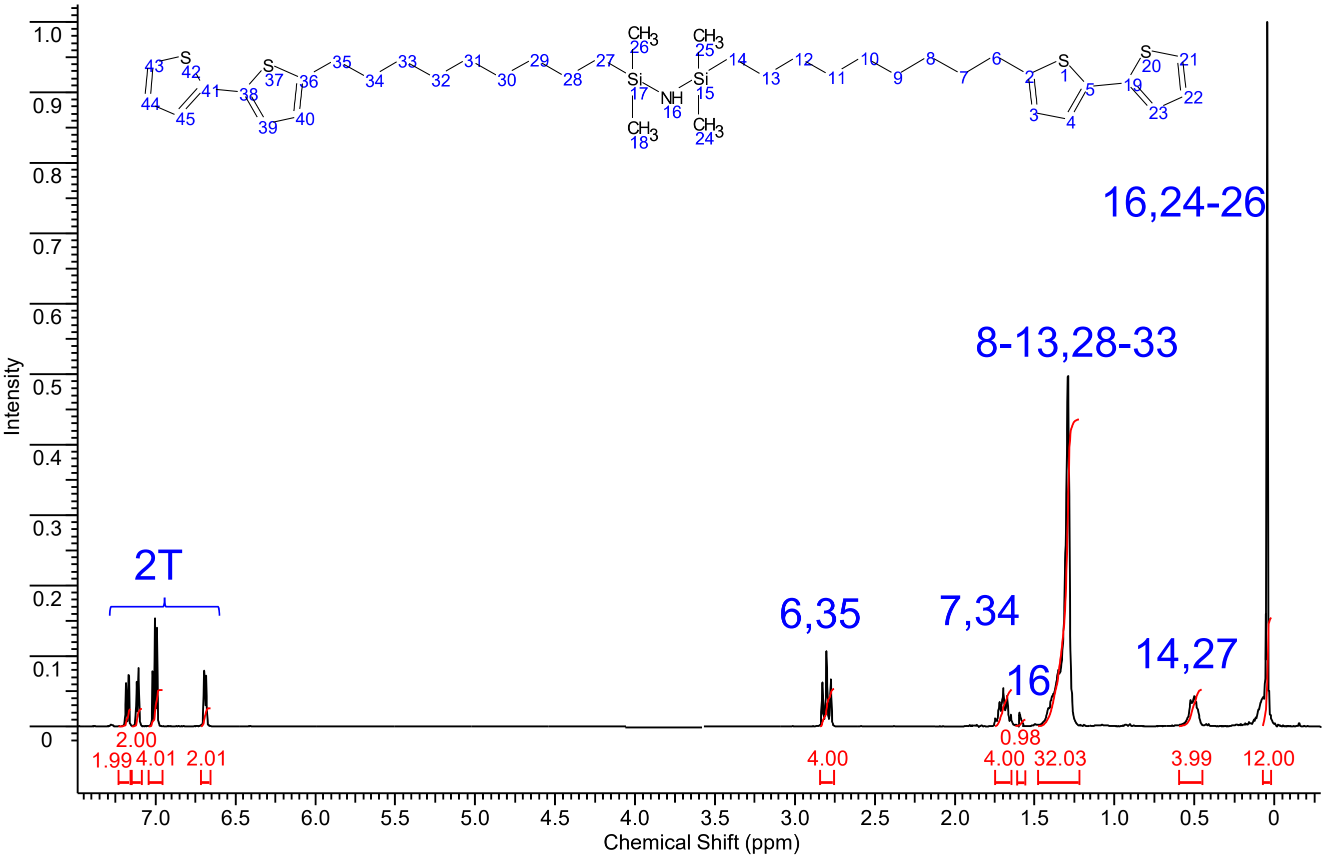

Figure S4. ^{1}H NMR spectrum of **NH(Si-Und-2T)$_2$** in $CDCl_3$.

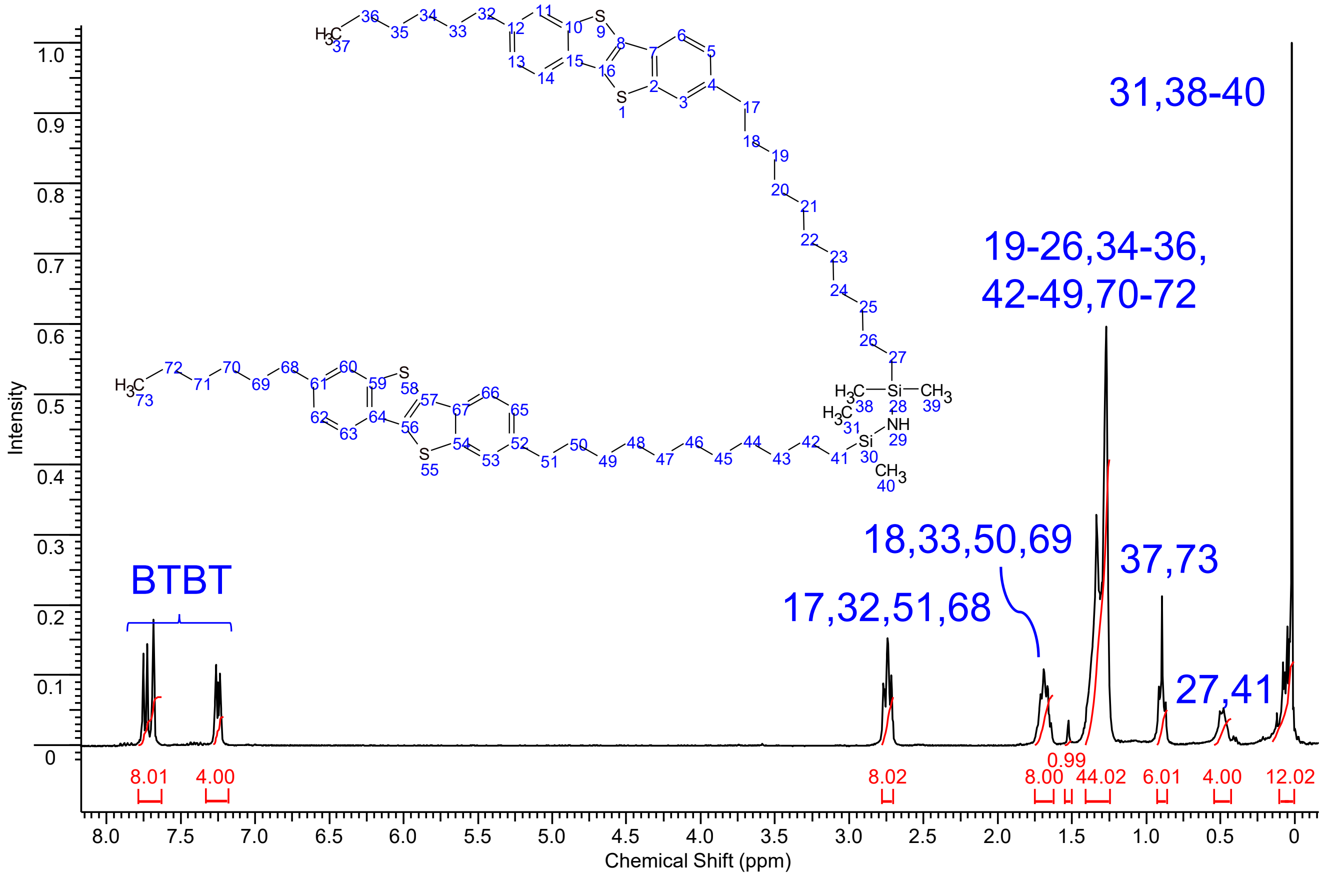

Figure S5. ^{1}H NMR spectrum of **N(Si-Und-BTBT-Hex)$_2$** in $CDCl_3$

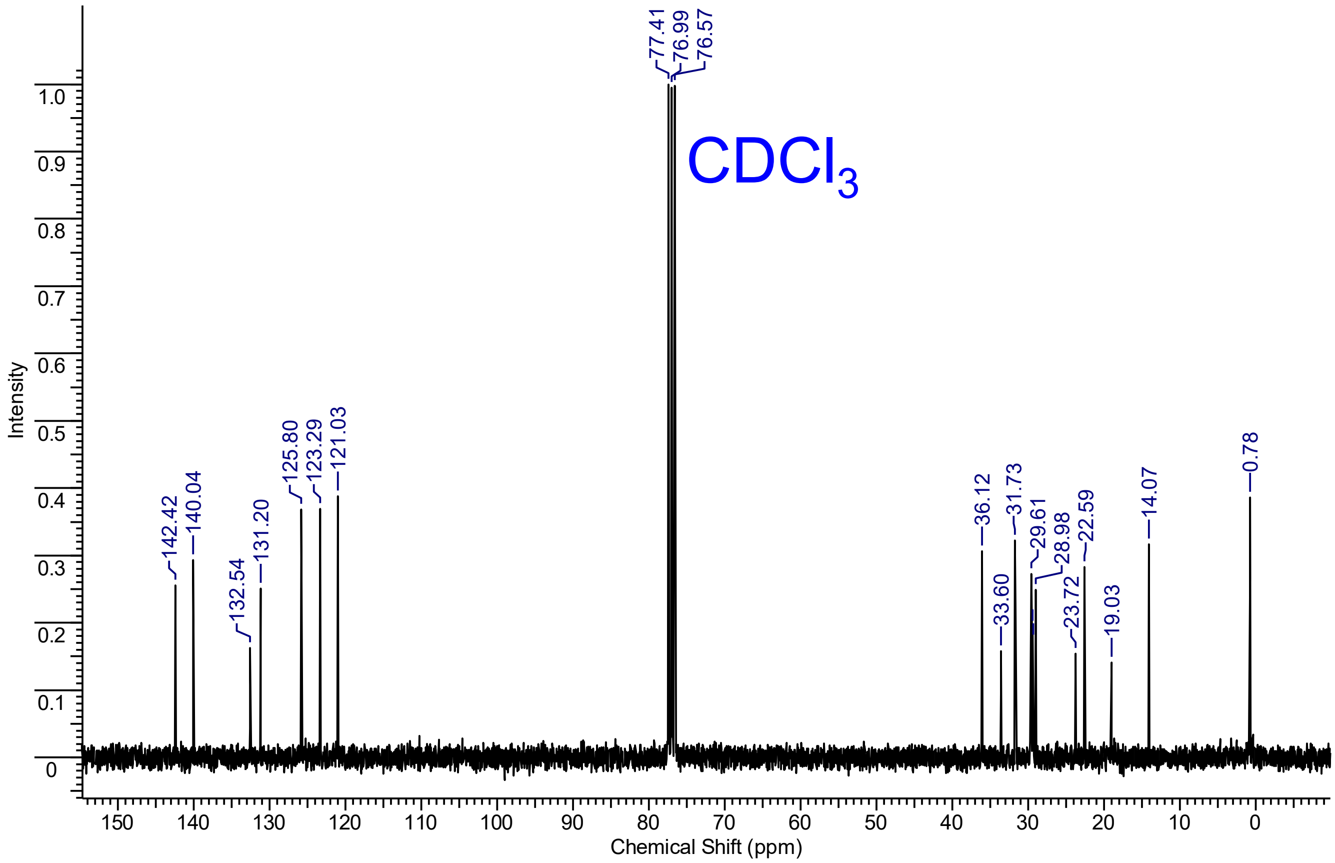

Figure S6. ^{13}C NMR spectrum of **HN(Si-Und-BTBT-Hex)$_2$** in $CDCl_3$

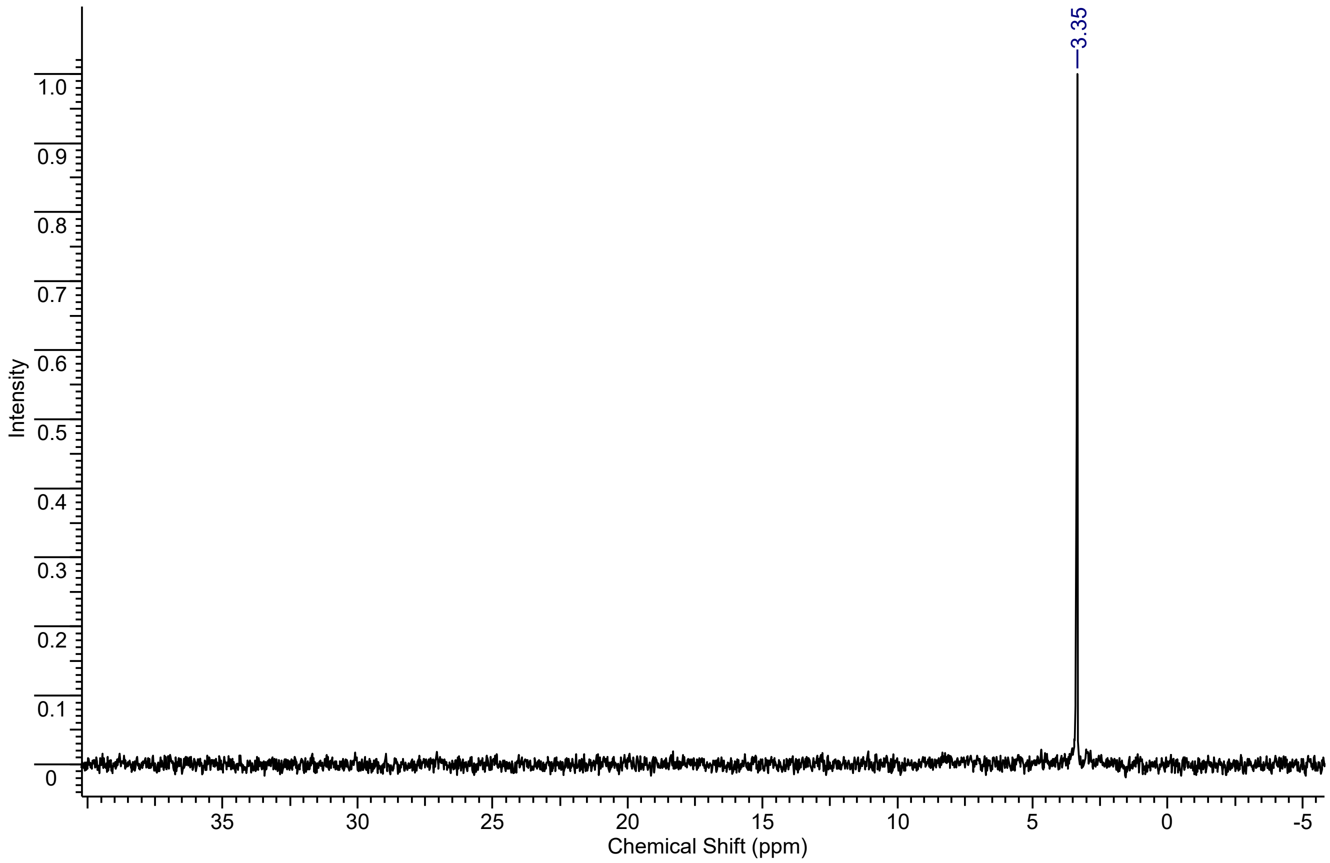

Figure S7. ^{29}Si NMR spectrum of **HN(Si-Und-BTBT-Hex)$_2$** in $CDCl_3$

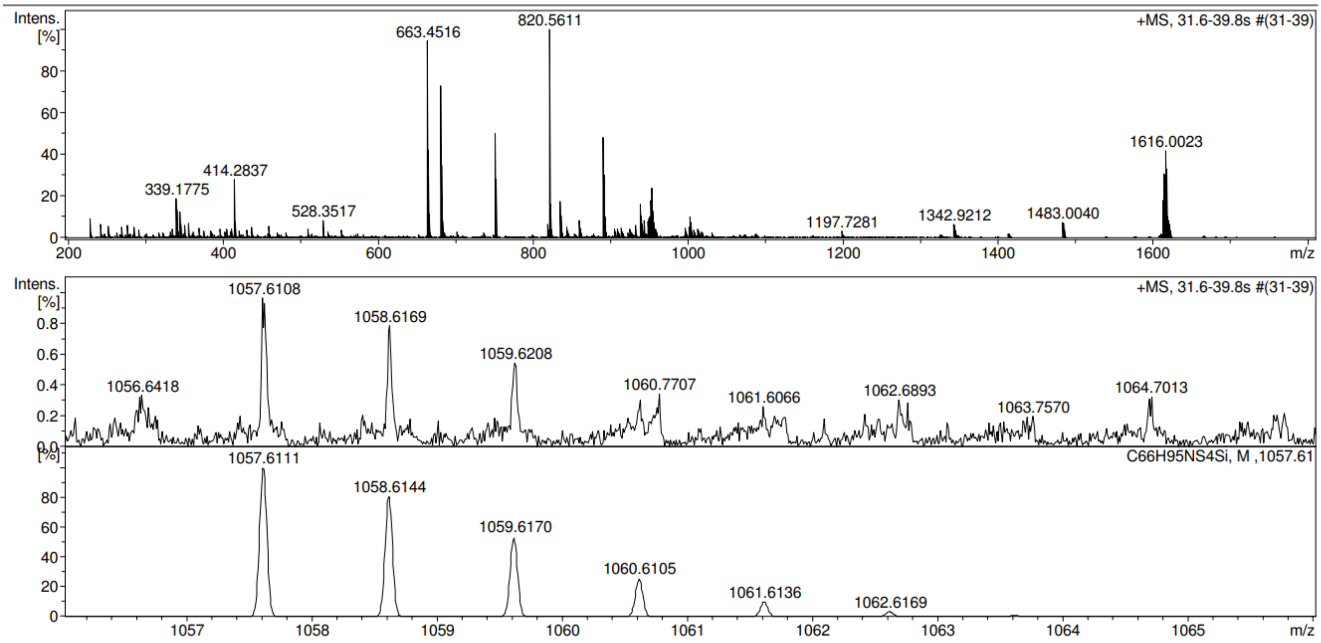

Figure S8. MALDI TOF spectra of **HN(Si-Und-BTBT-Hex)$_2$**

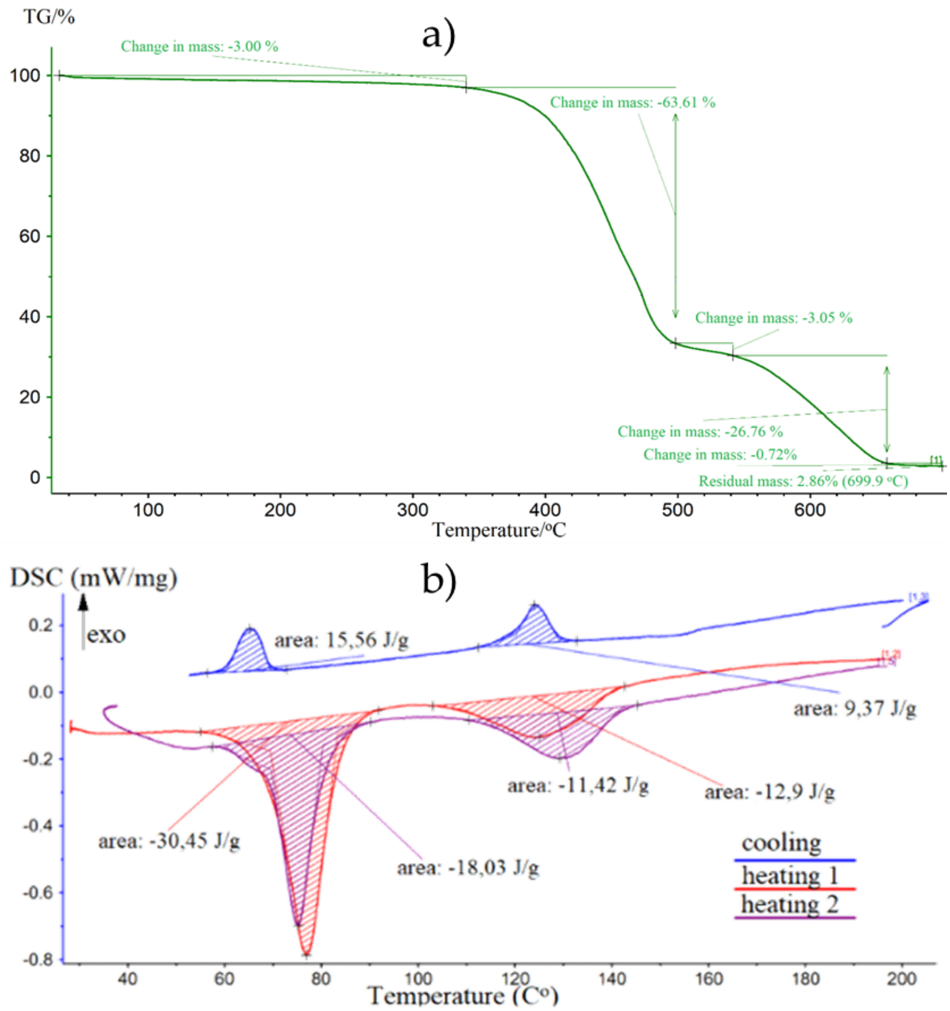

Figure S9. TGA of **HN(Si-Und-BTBT-Hex)$_2$** in the air (a); DSC curves for **NH(Si-Und-BTBT-Hex)$_2$**; the red curve is the 1st heating, the blue one is cooling, and the purple one is the 2nd heating (b)

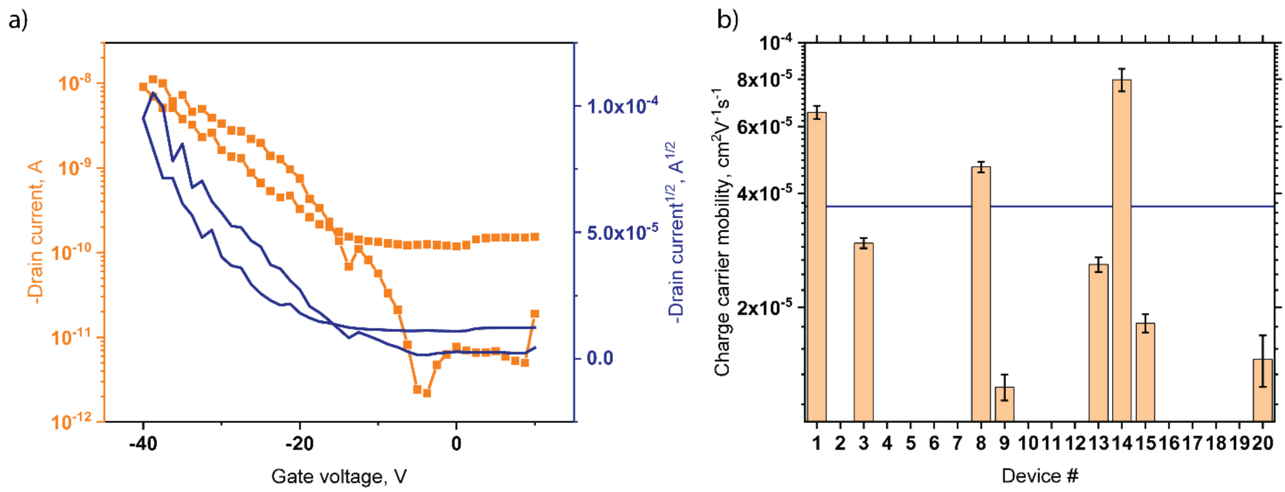

Figure S10. A typical transfer curve (a) and charge carrier mobility distribution (b) for the LB OFET based on silazane dimer **HN(Si-Und-BTBT-Hex)$_2$**

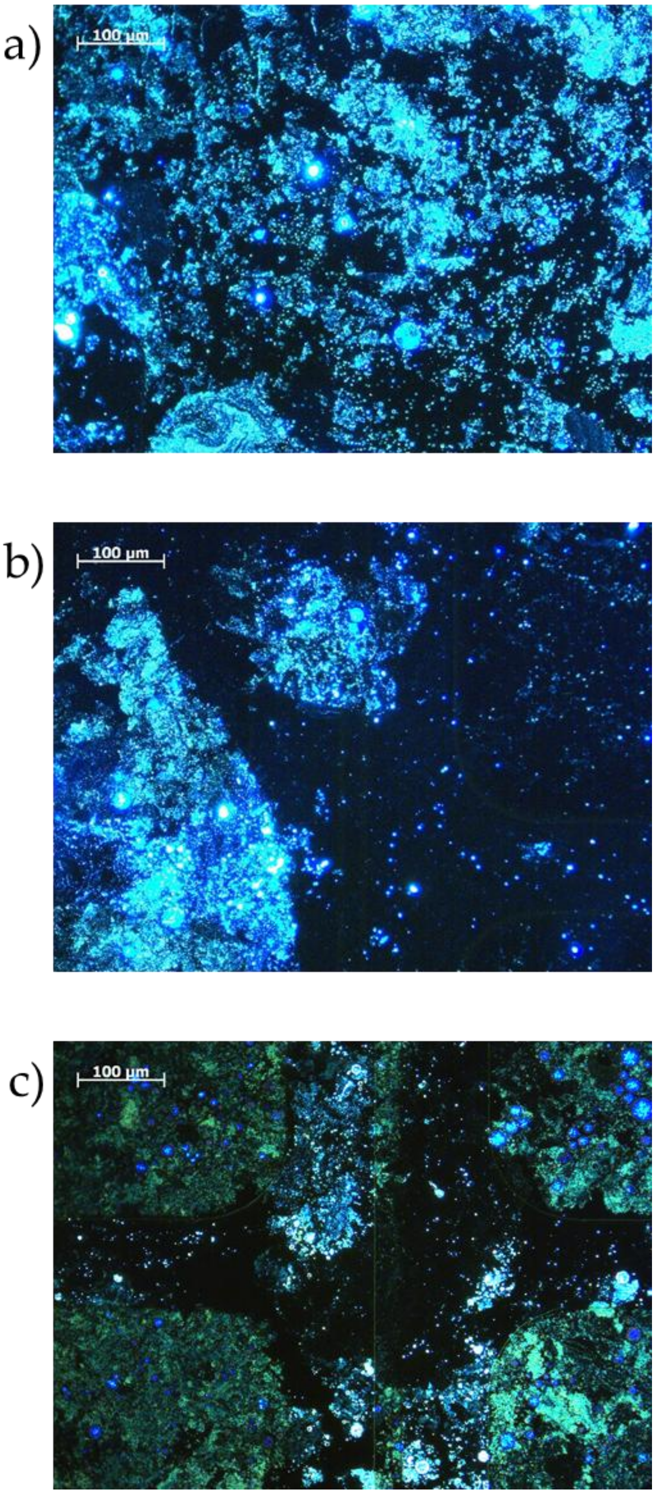

Figure S11. A film cracking during transfer of silazane dimer **HN(Si-Und-BTBT-Hex)$_2$** from water surface via LB technique: a) 0.33 g L^{-1}, b) 0.5 g L^{-1}, c) 1 g L^{-1}.

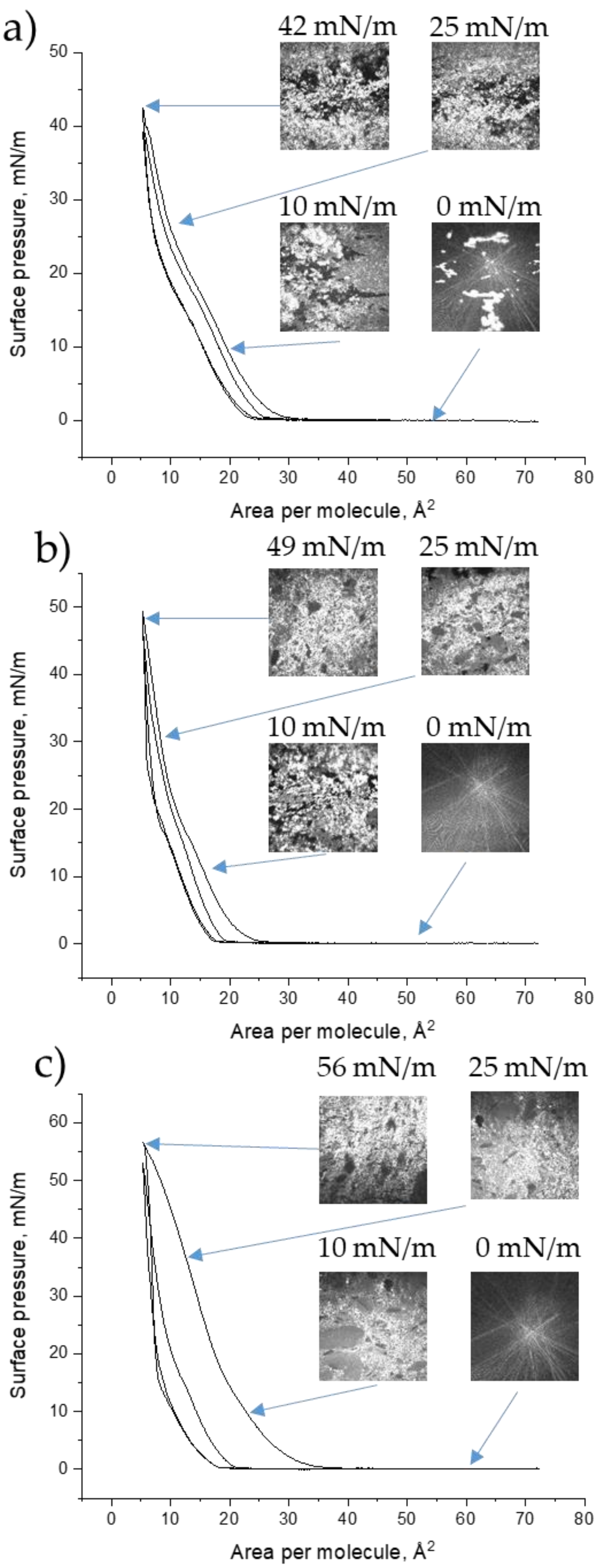

Figure S12. Langmuir isotherms for silazane dimer **HN(Si-Und-BTBT-Hex)$_2$** (compression-decompression-compression cycle) with the Brewster angle microscopy images obtained at the indicated surface pressures: a) 0.33 g L^{-1}, b) 0.5 g L^{-1}, c) 1 g L^{-1}.
